# Studies on the Digital Inclusion Among Older Adults and the Quality of Life—A Nanjing Example in China

**DOI:** 10.3389/fpubh.2022.811959

**Published:** 2022-05-12

**Authors:** Hui Yang, Hongtu Chen, Tianshu Pan, Yiran Lin, Ying Zhang, Honglin Chen

**Affiliations:** ^1^Department of Sociology, Minzu University of China, Beijing, China; ^2^Department of Global Health and Social Medicine Affiliate, Harvard Medical School, Boston, MA, United States; ^3^School of Social Development and Public Policy, Fudan University, Shanghai, China; ^4^College of Public Administration, Nanjing Agricultural University, Nanjing, China; ^5^Dongfang College, Zhejiang University of Finance and Economics, Jiaxing, China; ^6^Department of Social Sciences, University of Eastern Finland, Kuopio, Finland; ^7^Department of Social Work, Fudan University, Shanghai, China

**Keywords:** digital inclusion, quality of life, multinomial logit model, older adults, Nanjing

## Abstract

Digital inclusion can bridge the digital divide and reduce the social exclusion of older adults, yet it is understudied in China. This research examined factors influencing the digital inclusion of older adults in China and the relationship between digital inclusion and quality of life. Data collected from 312 older people (*M* = 69.6 years old) in Nanjing were included in a multinomial logit model to tackle these questions. Their attitudes toward technology were the most significant factor predicting their digital inclusion. Other factors included party affiliation, living situation, personal average monthly income, occupation, and capacity for instrumental activities of daily living (IADLs). This study shows digital inclusion has a direct impact on quality of life. It also serves as an intermediate variable that affects older people's attitudes toward technology and their IADL capacities. Most importantly, digital inclusion promotes social integration of older adults and improves the quality of their lives. Hence, it should not be ignored. Older people's attitudes toward technology are one of the keys to promoting their digital inclusion.

## Introduction

The two great trends of our era are the internet and the aging of our population. The former is becoming a tool to cope with the latter and a means for building a smart aging society. Internet support can help improve services for older adults, effectively target their needs, and increase competencies for managing seniors (1). The internet has revolutionized the means of social participation among older people (2). It has also become a pathway for improving their physical and mental health and their sense of wellness and satisfaction.

However, a smart aging society will face many difficulties. The elderly may have lower economic status as China's economy, cost of living and income levels grew dramatically over the last 20 years. Some face the difficulties associated with declining strength and sensory functions, less access to technology and learning, and may be more risk averse. Compared with younger people, they are less likely to be exposed to digital information and communication technology (ICT). Thus, they are particularly vulnerable to missing out on the benefits of technology and finding themselves lost to the “digital divide.” This problem should never be taken lightly. If left unattended, this could lead to a disconnect between older people and society (3). Closing the digital divide, reducing social exclusion through informatization, facilitating digital inclusion, and achieving greater inclusion in a harmonious society have become important goals. However, the study of digital inclusion among older adults in China is in its infancy. This paper provides some initial responses to the following questions: what factors affect digital inclusion among older adults in China? Does digital inclusion of older adults play any role in their quality of life?

## Literature Review and Theoretical Framework

Digital inclusion is the process of closing the digital divide (4). This means the “individual and community are free from barriers to access to information through information and communication technology, thereby effectively participating in every aspect of knowledge society and economic development, and obtaining social benefit according to their will and capacity” (5).

In empirical studies outside China, digital inclusion is closely related to many factors. The Multidimensional Explanatory Conceptual Model of Information Society Inequality proposed by De Haan (6) helps us clarify the levels and relationships among various influencing factors. On the basis of Rogers' innovation diffusion theory and Coleman's capital theory, combined with many previous empirical research results, De Haan classified the influencing factors of the digital divide into structural and individual factors and argued that these different dimensions did not act independently on individual IT access, but worked together to form uneven social, material, cognitive (i.e., human capital), and time resources (counted as a type of material resources), which ultimately led to unequal access to technology (6). Individual characteristics include age and generation, gender, ethnicity and race, intelligence, and personality, and opportunity structures, including family status, education system, and labor market.

Drawing on this multidimensional explanatory model of information society inequality, combined with the latest relevant studies in and outside China and the characteristics of the Chinese older adult population, we can reinterpret the influencing factors of digital inclusion of Chinese older adults from two perspectives: personal characteristics and opportunity structure.

Regarding personal characteristics, gender and age are the basic variables that have received more attention, and most studies found that gender differences are also prevalent among the older adult population, where men have higher intention to use the internet than women (7). Old-old adults are also more limited in their use of the internet due to a higher degree of physical decline than young-old adults (8); therefore, age may reflect its consequences, such as the impact of daily living abilities, and many studies have shown that health status is a key factor influencing internet use among older adults, with healthier older adults being more likely to use the internet (9). Older adults with impairments in vision, hearing, and finger dexterity are less likely to use the internet (10). In turn, all instrumental activities of daily living (IADLs) may also be supported by technology (11). Accordingly, the first hypothesis of this study is proposed.

### H1.1: IADLs Are Positively Associated With Digital Inclusion

According to the Multidimensional Explanatory Conceptual Model, individual factors of ability such as intelligence and human capital would predict greater access to technology. In terms of structural characteristics, family, school, and workplace are often considered the context for accessing various resources. At the family level, for older adults, marital status and residential status (12) influence their internet use behavior through device and technical support. These two variables affect not only household possession and access to resources, but also the individual's ability to access technology resources and use support from the household. It has been shown that living with children or partner has a positive effect on older adults' learning to use the internet (12) and that widowed and divorced older adults use the internet significantly less frequently than unmarried and married groups (9). Accordingly, the following hypotheses were proposed.

### H1.2: Marital Status Is Positively Related to Digital Inclusion

The Multidimensional Explanatory Conceptual Model would further present the next hypothesis on CPR-residence as residential status (12) influences device support and technical support.

### H1.3: Coresident Living Situation Is Positively Associated With Digital Inclusion

The Multidimensional Explanatory Conceptual addresses education as a pathway to enhance digital skills, but it may also be a response to cognitive ability (antecedents) and occupation (consequences) for older adults born before the internet age (13). Still, a large body of research suggests that educational attainment is consistently associated with internet use behavior among older adults (13) and that older adults with higher educational attainment are more literate and will engage more in information-sending and news-reading activities (14). Therefore, the model supports the following hypothesis.

### H1.4: Level of Education Is Positively Related to Digital Inclusion

Older adults in the non-networked generation are more likely to develop their ICT ownership and digital skills in the workplace. Not only is the workplace a key arena for individuals to develop their social networks, but it is also an important place to obtain material resources (e.g., income, electronic devices). The former implies that others in the social network can influence the purchase and use behavior of personal digital products by providing IT support, creating an atmosphere of acceptance of new products and reducing uncertainty about the purchase of new products (6). The latter influences the acquisition of personal digital products; occupations with higher social status are more likely to have access to the internet that provides access to a wealth of information. Some studies have shown that older adults who engaged in physical and mental labor before retirement differed significantly in terms of the difficulty of learning to use the internet, their intention to use it, and the length of time spent online (15). In addition to the binary division between physical and mental labor, party membership is also usually associated with higher socioeconomic status in China, and these individuals have unique advantages in terms of career advancement and expanding their network resources, especially those with cadre status, and as such, these older adults perform better in terms of using new media technologies (16). The following hypothesis is therefore proposed.

### H1.5: Retirement From Occupation Is Negatively Associated With Digital Inclusion

Given the importance of party affiliation to access to community resources, the Multidimensional Explanatory Conceptual Model would predict the added Social Capital and structural advantages of being engaged in party-sponsored events would positively affect access to technology (6).

### H1.6: Party Affiliation Is Positively Related to Digital Inclusion

Although income, education level, and occupation are seen as three important variables for measuring socioeconomic status and there are correlations among the three, income still has an independent role after controlling for the education variable, due to the fact that economic factors mainly govern older adults' ability to purchase equipment and maintain its operation, which is closely related to whether they use the internet (17). However, some scholars have argued that income is not related to ICT use among older adults (18). Therefore, we examined income as well and proposed the following hypothesis.

### H1.7: Personal Average Monthly Income Is Positively Associated With Digital Inclusion

We examined both these personal characteristics and opportunity structure factors in the context of basic demographic variables. In addition, the rational behavior model suggests that attitudes are the positive or negative feelings that people have about engaging in a target behavior and are determined by their primary beliefs about the outcome of the behavior and their estimates of the importance of that outcome. An individual's behavioral intentions are influenced by behavioral attitudes, and attitudes ultimately have a large impact on individual behavior by acting on behavioral intentions. Positive or negative attitudes toward technology and its consequences also are an important reason for the digital divide among people, because attitudes are the combined result of the interaction of factors such as the uneven geographical development of ICT technologies, individual personalities, socioeconomic activities, demand for the internet, and product characteristics (19–21). Many empirical studies have also shown that positive attitudes toward digital technologies in the age of consultation and networking increasingly affect the normal functioning of life and that lack of interest can be considered a barrier to access to resources, participation in the community, and a cause of the digital divide among people (16, 19). Therefore, we incorporated the consideration of attitudinal elements and proposed the following hypothesis:

### H1.8: Older Adults' Positive Attitudes Toward Technology Are Positively Associated With Digital Inclusion

International studies have proven the positive effect of digital inclusion on older people's physical, mental, and spiritual wellness. Learning about and using ICT and the internet can improve older people's cognitive ability (22); enhance their relationships with family and friends; reduce their sense of loneliness (23); strengthen their sense of self-efficacy (24), independence, and self-growth; and promote their social integration (24). These all increase their community satisfaction (18) and quality of life (25). Studies of older Chinese people and the relationship between digital inclusion and their quality of life is relatively lagging. To study these issues, we proposed another set of hypotheses. This second set of hypotheses involved the relationship between the digital inclusion of older adults and their quality of life.

### Hypothesis 2: Digital Inclusion of Older Adults Affects Their Quality of Life

Among factors that may affect the quality of life of older adults, scholars have paid extensive attention to basic demographic variables such as gender, age (26), income (27), region, occupation (28), education level (29), marital status (30), living status (31), and physical and mental health (32), proving that they are closely related to quality of life in old age. Therefore, this study incorporated some of the key factors in Hypothesis 1 as control variables to more clearly observe the independent effect of digital inclusion.

**Figure 1:**
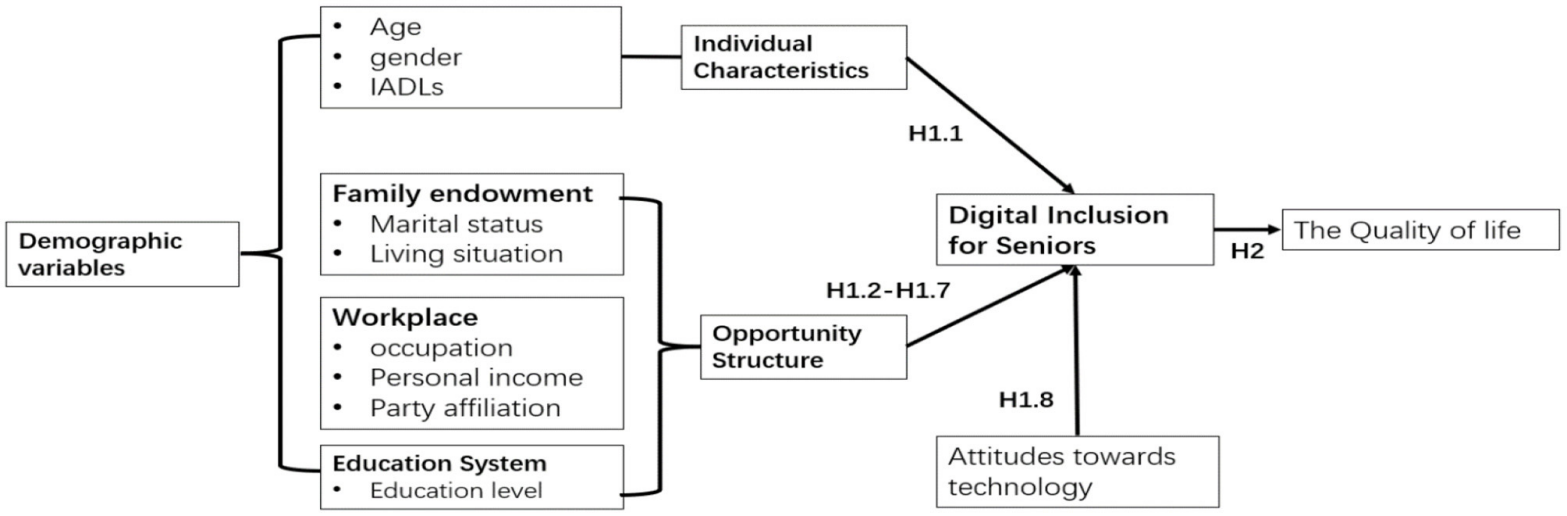
Theoretical framework of this study.

The theoretical framework of the study is shown in Figure 1.

## Methods

### Sampling and Data Collection

Nanjing was selected as the survey site because it is an important city with the dual characteristics of being a “digital city leader” and “aging society.” On one hand, Nanjing's population of adults aged 60 or older reached 10.04% of the total population as early as 1990, indicating that Nanjing has become an aging society. According to the latest census results, the population aged 60 or older in Nanjing accounted for 18.98% of the total population in 2020, an increase of 5.23% compared with 2010, and the population aged 65 or older accounted for 13.70%, an increase of about 4.52% (33). The aging of the population in Nanjing is showing a continuous acceleration, which means that Nanjing has been and will be an aging society for a long time (34). On the other hand, as early as 2006, when the State Council issued the 2006–2020 National Informatization Development Strategy, Nanjing proposed the goal of building a “digital Nanjing.” In recent years, the production and sales rate of electronic products in Nanjing has been high, and the manufacturing industry of communication equipment, computers, and other electronic equipment has maintained strong growth. This rapid informatization has attracted many smart pension projects to be stationed and implemented there. Nanjing has outstanding advantages nationwide in both the information and communication industry and the development of the “internet add pension industry,” and it is a pioneer of smart city construction (35). This research focused on adults aged 60 or older in Nanjing as its survey participants.

Due to the survey's many questions and the challenges they posed to older people, the survey staff assisted the respondents if they needed help. Interviewers were recruited from among postgraduate students in social work and anthropology with survey experience. The study team provided systematic training on the questionnaire survey for the interviewers before the investigation began. The selection of study objects followed the principle of simple random sampling, stratified sampling, and systematic sampling methods. The process was as follows: (a) Random communities in Nanjing were selected; they were Mufu Community, Jiangwan Community, Baota Community, and Jinling Community. (b) The communities were categorized according to their economic status (public budget revenue and disposable income per capita) and geographic location—either downtown or not downtown community (city suburb). Of the four communities selected, three—Mufu, Jiangwan, and Baota—were in the downtown communities of Gulou and Jianye. Jinling is in the city suburb of Qixia. According to data from the *Information of the Senior Population and Report on the Development of Elderly Affairs in 2017 in Nanjing* by Nanjing Bureau of Civil Affairs, the calculated ratio of the older adult population in downtown to that in the city's suburbs is approximately 2:3. (c) Based on the general city-to-suburb ratio of the older adult population (2:3), equidistant sampling was conducted with the stratified sampling frame of residents aged 60 or older in the four selected communities. Based on the equidistant sampling, participants with the following criteria were excluded from the study: those unable to participate in the interview due to health conditions; those who did not currently live at their residential address in the community and whose current address was relatively far away; those whose spouses had been selected as participants; and those who declined to participate in the interview. (d) Given the difficulties of equidistant sampling, maximum effort was made to make the downtown-to-suburb ratio of the sample close to the real ratio. Of 350 questionnaires distributed, 341 were returned, of which 312 were valid. The number of valid responses met our expectations. The basic information of these survey subjects is shown in Table 1.

**Table 1:** Basic information of survey subjects.

**Variable**	**Category**	**Number of people**	**Percentage**	**Cumulative percentage**
Age	60–65	96	30.9%	30.9%
	66–70	99	31.8%	62.7%
	71–75	61	19.6%	82.3%
	76 and above	55	17.7%	100.0%
Gender	Female	180	57.9%	57.9%
	Male	131	42.1%	100.0%
Party	Non-partisan	201	68.6%	68.6%
affiliation	Member of a political party	92	31.4%	100.0%
Marital status	Unmarried	57	18.7%	18.7%
	Married	248	81.3%	100.0%
Living	Older adult living alone	32	10.5%	10.5%
situation	Older adult not living alone, not with young people living at home	155	51.0%	61.0%
	Older adult not living alone, with young people living at home	117	38.5%	100.0%
Level of education	Primary school and below (including illiterate)	74	23.8%	23.8%
	Junior high school	101	32.5%	56.3%
	High school and above	87	28.0%	84.3%
	Collage and above	49	15.8%	100.0%
Occupation	Manual labor	111	37.9%	37.9%
(current/before retirement)	Non-manual labor	182	62.1%	100.0%
Personal	Below 2,000	35	11.5%	11.5%
average	2,000–2,999	53	17.4%	28.9%
monthly	3,000–3,999	148	48.5%	77.4%
income	4,000 and above	69	22.6%	100.0%
Attitude	Negative	52	23.6%	16.7%
toward technology	Positive	168	76.4%	54.0%
IADLs		Average value 23.63		

### Measurement

According to our study's needs, the following content was included in the questionnaire: the table of digital inclusion and questions regarding the respondent's attitude toward the internet and information technology products, quality of life, health condition, and personal information. Among these inquiries, the respondent's attitude toward the internet and information technology products involved their “opinions on the internet” and “attitude toward products of information technology”; physical condition included disability status and capacities for IADLs; and personal information included social characteristics of gender, age, hometown, party affiliation, level of education, marital status, living situation, occupation, years of residence in the area, number of children, household monthly income, monthly income per capita, etc. The questionnaire was developed based on a questionnaire used by the research team of the University of Hong Kong in the study of digital inclusion in Hong Kong (36), which has been used in research in Shanghai with good reliability and validity (37, 38). The operationalized description of the key variables is as follows.

#### Digital Inclusion

The research team of the University of Hong Kong, which studied digital inclusion in Hong Kong with a sample of 2,596 participants including vulnerable groups (36), collected firsthand information regarding four dimensions of ICT: availability, affordability, usage, and knowledge level. They also established a comprehensive digital inclusion index. This was the first attempt among similar research efforts in China and around the world. It was also the starting point for further discussions of the digital divide. This study adopted this comprehensive digital inclusion index and made minor adjustments according to the situation of Nanjing respondents before data collection—for example, “percentage of elderly people who have used www.e123.hk.” Because this website is unique to Hong Kong, it was not suitable for mainland older adults to answer, and there was no equivalent website relevant to mainland China; thus, this question was removed in this study. The final table of measurements for our study included 15 indicators assessed via 17 questions; of which, two were follow-up questions if the respondent selected a certain option. The survey measured dimensions including internet access and usage, internet knowledge and skills, etc. Referring to the calculation method of the European Union, the ultimate personal value of digital inclusion was the average of all indicators.

#### Quality of Life

Among the various scales measuring the quality of life of older adults, CASP-19 is a widely used tool (39). It is a Likert scale of 19 questions developed to examine the status of older people in the United Kingdom. It has four domains: control, autonomy, self-realization, and pleasure. CASP-19 and its simplified versions, CASP-14 and CASP-12, have shown relatively high applicability in Europe, Taiwan, and mainland China (39). This study used revised quality of life scale for older adults created in 2009 (CASP-14), which features 14 sentences describing life or feelings about life (40). Scores were based on the four response options of “often,” “sometimes,” “rarely,” and “never.” The value for each question ranges from 1 to 4 points, and the final quality of life index is the average score of all dimensions.

#### Other Key Independent Variables

The operationalization of other key variables included: (a) Marital status, which involved the influence of spouses on the internet use of older adults and was coded as “married” or “unmarried” (single, widowed, divorced, or separated). (b) Living situation, which involved the influence of children on the digital inclusion of older adults: “living alone” (no young people in the home) and “not living alone” (young people in the home). (c) Level of education, which was divided into primary school and below, junior high school, high school or beyond, or college or beyond—colleges, universities, and postgraduate studies were merged into one type because higher education was not widely popularized in the 1940s and 1960s, when most of the current older adults were educated, and most college education referred to junior college (15). (d) Occupation before or during current retirement, which follows the method of Jiao (41). Considering that the occupational dispersion of middle-aged and older people in China is not very large, if occupations were divided into more types, it would lead to many empty cells, which may have affected the results of model estimation. Therefore, occupations were divided into two categories: manual labor, including farmers and workers, and non-manual labor, including management, professional and technical, office, and business service personnel. (e) Party affiliation, which refers to membership in the Communist Party of China and was treated as a yes-or-no dichotomous variable. (f) Economic level, in which those whose per capita annual household income was less than half of the sample median were considered to be in poverty—the “personal average monthly income” variable determined the approximate boundary of each group based on quartile, so that the population in each group was relatively evenly distributed. (g) Attitudes toward technology: this study used a self-assessment method, asking respondents to comprehensively describe their attitudes toward ICT technology. Samples containing positive words like “satisfied,” “support,” and “like” or neutral words like “double-edged sword” were coded as “positive attitude.” Negative responses such as “dissatisfied,” “unsupported,” “do not understand,” and “can't learn,” were coded as “negative attitude.”

## Statistical Methods

Each hypothesis was tested based on the level of measurement of the variables. Methods included correlation, ANOVA, independent samples T test, and multiple regression to determine the direction of the relationship of each independent variable with digital inclusion.

## Results

### Exploration of the Influencing Factors of Digital Inclusion

This set of hypotheses concerned the effect of basic demographic variables on digital inclusion in older groups. The tested variables were age, gender, IADLs, marital status, living situation, education level, current and preretirement occupation, party affiliation, personal average monthly income, and attitudes toward technology.

The independent variables and their relationship with digital inclusion were examined individually. Two methods were used: analysis of variance or independent-samples *t*-test. The independent variables related to the dependent variable were introduced into the multiple regression model to test whether the model could predict the relationship between the variables as hypothesized.

After exploring all potential independent variables and their relationship with digital inclusion one by one, we found party affiliation (*p* <0.01), living situation (*p* <0.05), education level (*p* <0.01), personal average monthly income (*p* <0.01), occupation (*p* < 0.01), IADLs (*p* < 0.05), and attitudes toward technology (*p* < 0.01) all had significant correlations with different levels of digital inclusion.

Multiple regression analyses of the independent variables related to digital inclusion were conducted to explore explanatory models of dependent variables. The recommended model for multiple regression analysis is as follows.

Digital inclusion is a linear function of education level, party affiliation, living situation, personal average monthly income, occupation, IADLs, and attitudes toward technology.

To ensure an optimal model of multiple linear regression analysis, hierarchical regression analysis was warranted to explore the contribution of each variable to the explanation of the dependent variable. The seven independent variables were put into the model in three steps: (a) demographic variables, including education level, party affiliation, living situation, personal average monthly income, and occupation; (b) IADLs; and (c) attitudes toward technology. The third model had the strongest power, explaining 31.9% of the variance in digital inclusion. The test results are shown in Table 2.

**Table 2:** Hierarchical regression analysis.

	**Variable**	**Step one**	**Step two**	**Step three**
Step one	**Personal characteristics**			
	IADLs	0.269^*^	0.207	0.128
Step two	**Opportunity structure**			
	Marital status		0.096	0.036
	Living situation		−0.216	−0.003
	Occupation (current/before retirement)		0.167	0.123
	Party affiliation		−0.029	0.067
	Personal average monthly income		0.261^*^	0.100
	Level of education		0.092	0.021
Step three	**Attitude toward technology**			0.479^***^
	F	5.790^*^	2.969^**^	5.155^***^
	R^2^	0.073	0.234	0.381
	ΔR^2^	0.060	0.155	0.307

* *p < 0.05*;

** *p < 0.01*;

*** *p < 0.001*.

Model 1.1, in which only one factor representing personal characteristics, IADLs, was introduced, was significant (Δ*R*^2^ = 0.060). This shows that digital inclusion was positively influenced by IADLs and the effect was significant. The higher the average monthly income of the individual, the higher their digital inclusion.

Model 1.2 introduced variables representing the three dimensions of opportunity structure, which increased the explanatory effect of the model regarding digital inclusion to 15.5% and was statistically significant. When these variables were added, only personal average monthly income had a significant positive effect on the dependent variable, and IADLs were not statistically significant in the model. Nevertheless, IADLs in Model 2 played a role in digital inclusion.

In Model 1.3, all predictor variables were used simultaneously. The multivariate regression coefficient was 0.617 (*R*^2^ = 0.381) and adjusted *R*^2^ was 0.307, implying that the three categories of individual characteristics, opportunity structure, and attitudes toward technology together explained 30.7% of the variance in digital inclusion. Among all predictor variables, the *t*-test of the unstandardized regression coefficient (*b*) of the newly added “attitudes toward technology” variable shows that it contributed to competence in digital inclusion. This indicator contributed most of the explanatory power (*b* = 0.163, β = 0.479, *t* = 3.988, *p* < 0.001), suggesting it is a critical variable for digital inclusion. The more an older adult held positive attitudes toward technology, the higher their digital inclusion.

Therefore, digital inclusion among older Chinese people is a linear function concerning level of education, party affiliation, living situation, personal average monthly income, occupation, IADLs, and attitudes toward technology.

As shown in Table 3, the result of the analysis of variance was *F*_(75)_ = 5.155, *p* < 0.001, indicating the integration of all indicators effectively predicted digital inclusion. The hypothesis of linear distribution, normal distribution error, and uncorrelated error were properly tested and considered. Judging from inclusion (>0.10) and variance inflation factor (<5) values, these related variables did not produce collinearity problems. In the final model, the introduction of attitudes toward technology reduced the effect of personal average monthly income, a variable of statistical significance, on digital inclusion. Thus, in the complete model, demographic variables had no influence on the dependent variable. Attitudes toward technology played a major role and positively correlated with digital inclusion. Other variables played a role based on attitudes toward technology.

**Table 3:** Final model statistics of digital inclusion.

	**B**	**SED**	**Beta**	***T***	***P***	**Tolerance**	**VIF**
(Constant)	0.241	0.245		0.986	0.328		
IADLs	0.012	0.010	0.128	1.255	0.214	0.889	1.125
Marital status	0.013	0.048	0.036	0.275	0.784	0.546	1.831
Living situation	−0.001	0.027	−0.003	−0.020	0.984	0.480	2.083
Occupation (current/before retirement)	0.033	0.028	0.123	1.156	0.252	0.818	1.222
Party affiliation	0.018	0.030	0.067	0.588	0.558	0.719	1.390
Personal average monthly income	0.015	0.016	0.100	0.909	0.367	0.762	1.312
Level of education	0.003	0.017	0.021	0.185	0.854	0.689	1.452
Attitude toward technology	0.163	0.041	0.479	3.988	0.000	0.641	1.560

*Durbin-Watson: 1.651; F_(75)_ = 5.155, P < 0.001*.

### Relationship Between Digital Inclusion and Quality of Life

The second set of hypotheses primarily examined the relationship between digital inclusion of older adults and their quality of life.

Predictor variables significantly related to the quality of life were incorporated to conduct a multiple regression analysis. Demographic variables significantly related to the quality of life of older adults included living situation, *F*_(292)_ = 4.926, *p* = 0.008, and marital status, *t* = −4.146, *p* < 0.01. In addition, digital inclusion, *r*_(116)_ = 0.322, *p* < 0.001; disability status, *t* = −3.438, *p* = 0.001; attitudes toward technology, *t* = −3.365, *p* = 0.001; and IADLs, *r*_(296)_ = 0.334, *p* < 0.001, all had correlations of different levels of significance with the dependent variable.

Multiple regression analysis was performed on these variables related to quality of life to explore the explanatory model of the dependent variable. The recommended model after multiple regression analysis is as follows.

Quality of life is a linear function of marital status, living situation, IADLs, disability status, attitude toward technology, and digital inclusion.

In this model as shown in Table 4, the tolerance of each variable was greater than 0.10, the VIF was <5, and the Durbin-Waston index was 1.670, indicating no serious collinearity problem. The analysis of variance of the model was *F*_(76)_ = 4.313, *p* < 0.005, indicating the combination of independent variables could effectively predict the quality of life of older adults.

**Table 4:** Regression analysis of influencing factors of quality of life.

**Model 2.1**	**B**	**SED**	**Beta**	**T**	**P**	**Tolerance**	**VIF**
(Constant)	−0.519	0.978		−0.531	0.597		
Marital status	0.083	0.195	0.056	0.428	0.670	0.603	1.659
Living situation	0.028	0.108	0.037	0.262	0.794	0.533	1.876
IADLs	0.061	0.042	0.164	1.433	0.156	0.799	1.252
disability status	0.260	0.175	0.166	1.483	0.143	0.828	1.207
Attitude toward technology	−0.224	0.152	−0.181	−1.480	0.143	0.694	1.441
Digital inclusion	1.757	0.457	0.460	3.849	0.000	0.730	1.371

*Durbin-Watson: 1.670; F_(76)_ = 4.313, P = 0.001*.

As the results show, when all predictors were used at the same time, the multiple regression correlation coefficient (*r*) was.520 (*R*^2^ = 0.270) and the adjusted *R*^2^ was 0.207. That is, all independent variables together explained 20.7% of the variation in quality of life. Among the predictors, only digital inclusion (*p* < 0.001) was statistically significant when acting together with the rest. Its beta value was the largest among all independent variables, i.e., digital inclusion contributed most of the explanatory power to the prediction of quality of life. The higher the digital inclusion of older adults, the higher their quality of life. The other indicators enhanced, to a certain degree, the significance of the overall model.

Therefore, does digital inclusion significantly explain quality of life? Regression analysis was performed to test Hypothesis 2.

Digital inclusion alone was very significant, *F*_(115)_ = 13.196, *p* < 0.01, Δ*R*^2^ = 0.096, in predicting quality of life. The unstandardized regression coefficient (*b* = 1.209) shows that for every 1-unit increase in digital inclusion, the quality of life of older adults increased by 1.209 units. The regression coefficient should be equal to the slope of its best-fitting model. The *R*^2^ value shows digital inclusion explained 9.6% of the variation in quality of life.

To further clarify the actual effect of digital inclusion on the quality of life of older adults, some demographic variables significantly related to quality of life in this study were then included as control variables to conduct a hierarchical regression test. Demographic variables that acted as control variables included living situation, *F*_(292)_ = 4.926, *p* = 0.008, and marital status, *t* = −4.146, *p* < 0.01.

Statistics show, after controlling for variables, digital inclusion had a significant, positive correlation with the quality of life of older adults (β = 0.320, *p* = 0.001). The higher the digital inclusion of older adults, the higher their quality of life. The overall model was significant, *F*_(111)_ = 4.520, *p* = 0.005, and 8.7% of the variation in quality of life can be ascribed jointly to digital inclusion and demographic variables. Yet when acting together, demographic variables had little effect on the quality of life and were not statistically significant. Only digital inclusion played a major role in the quality of life. Hypothesis 2.2 was thus verified. See Table 5 for details of data.

**Table 5:** Hierarchical regression analysis of quality of life and digital inclusion.

	**Variable**	**Model 3.1**	**Model 3.2**
**Step one**	**Demographic variables**		
	Marital status	0.162	−0.086
	Living situation	−0.069	0.050
Step two	**Digital inclusion**		0.320^**^
	*F*	1.169	4.520^**^
	R^2^	0.021	0.112
	ΔR^2^	0.003	0.087

** *p < 0.01*.

In addition, through the hierarchical regression analysis of influencing factors of quality of life, we found the introduction of digital inclusion caused a decrease in the beta value of two variables: attitudes toward technology and capacity for IADLs. This suggests digital inclusion may have a mediating effect on the quality of life.

In this study, the attitude toward technology and IADLs were important factors in the mental wellness and physical health of older adults, respectively. The internet plays a key role in promoting people's social connections and strengthening social support networks. Digital inclusion, therefore, is considered an important method to bring in social support. After reviewing the literature, we found the relationship among psychological factors, health status, social support, and quality of life is complex and the influential mechanisms of the first three variables on quality of life varied.

To further explore the mediating effect among digital inclusion, attitudes toward technology, and quality of life, further hypotheses were proposed based on our literature review:

**Hypothesis 3.1: Digital inclusion is a mediator between attitudes toward technology and quality of life**.
**Hypothesis 3.2: Attitudes toward technology is a mediator between digital inclusion and quality of life**.
**Hypothesis 3.3: Digital inclusion is a mediator between IADLs and quality of life**.

The three-step regression showed digital inclusion was a mediator between attitudes toward technology, IADLs, and quality of life.

Specifically, digital inclusion had a full mediation effect between attitudes toward technology and quality of life; attitudes toward technology predicting quality of life: β = 0.274, *p* < 0.01; attitudes toward technology predicting digital inclusion: β = 0.138, *p* <0.01. Attitudes toward technology and digital inclusion jointly predicted quality of life; digital inclusion: β = 1.175, *p* < 0.01; attitudes toward technology: β = −0.094, *p* = 0.519. This shows the attitudes of older adults toward technology affected their quality of life by influencing digital inclusion.

Digital inclusion mediated between IADLs and quality of life; IADLs predicting quality of life: β = 0.124, *p* < 0.01; IADLs predicting digital inclusion: β = 0.023, *p* < 0.05. IADLs and digital inclusion jointly predicted quality of life; digital inclusion: β = 1.127, *p* < 0.05; IADLs: β = 0.082, *p* < 0.05. This test shows some aspect of IADLs had an indirect impact on quality of life through digital inclusion, whereas the other part had a direct impact on the quality of life.

## Discussion and Conclusion

This study found that the degree of digital inclusion was affected by six factors, including party affiliation, living situation, personal average monthly income, occupation, IADLs, and attitudes toward technology. Attitudes toward technology accounted for a large proportion of the explanatory power of the model of digital inclusion, indicating that these attitudes are an important variable affecting the digital inclusion of older adults. This is similar to the findings of Correa and Pavez (19). One possible reason is that older adults who have a positive attitude toward technology are more interested in ICT and conduct activities through the internet more frequently. Today, older adults generally have fear and anxiety when facing technology products, so their attitude toward technology has a particularly significant impact on their digital inclusion.

In addition, digital inclusion significantly contributed to quality of life; when combined with variables such as demographic variables and attitudes toward technology, it still contributed most of the explanatory power to quality of life, and the impact was significant. This study also found that digital inclusion is a mediator that affects quality of life through older people's attitudes toward technology and capacity for IADLs. Older adults' attitudes toward technology affected their digital inclusion, whereas digital inclusion affected their quality of life. The capacity for IADLs of older adults played a role in their quality of life through digital inclusion, and it directly affected their quality of life in certain aspects. This is similar to the findings of Damant et al. (42), who found that healthy older people were more likely to benefit from ICT services and that ICT had a significant impact on their quality of life. More studies have shown that the physical health of older adults is the primary factor affecting their quality of life (43). These results are similar to those of the outcome analysis of mediation effects in our study.

Regarding the analysis of the impact of digital inclusion on quality of life, many domestic and foreign studies have drawn similar conclusions, proving that ICT can effectively improve the quality of life of older adults in different dimensions, including cognitive ability, social integration, and community satisfaction (18, 22, 24, 44), and these benefits seem to be related to developing a network of support from the internet (45, 46). The internet promotes communication between older adults and family, relatives, and friends; reduces loneliness; and enhances self-efficacy (24). It plays a role in social bonding, creating a connection between their world (where they are fairly isolated) and their family (including grandchildren), thereby effectively improving their quality of life (47). ICT has proven to be a new resource in both logical speculation and empirical research, and possession of digital resources yields independent and significant advantages in many fields (6). But is this advantage only strengthening the social capital of older adults in an advantageous socioeconomic position, thereby expanding the original social inequality, or is it compensating for the social capital of disadvantaged older groups and helping reduce social exclusion? There is no unified conclusion in the academic world. However, this study gives us a very important hint that in today's world of “active aging,” digital technology is becoming an important medium for creating an excellent environment that supports older living and a social climate that promotes healthy aging. In the process of technological progress and social development, inclusion of the older adult population should be promoted.

Therefore, ICT has become a new pathway for wellbeing among older adults. In previous studies, factors affecting their were roughly divided among demographic, family, cultural, economic, and social domains. Of those, social factors mainly consisted of social capital, social support, social participation, socioeconomic status, etc. They were interconnected and interacted with each other. All had a significant impact on older adults' quality of life. The positive effect of having a social network and social support are particularly prominent. With the information age, the internet has rapidly become a means of stabilizing and broadening social connections that strengthen social support. However, older adults are “digital refugees”; they are on the wrong side of the digital divide. This is hindering the possible improvement of their quality of life. Previous studies have revealed quality of life can be improved, and social support provided through modern tools should not be brushed aside. Digital inclusion is now an important carrier for older adults. For certain groups, it has supplanted traditional social factors that affect quality of life. It has become a new way to promote the social integration of older adults.

As this study reveals, attitudes toward technology are an important consideration. Changing those attitudes may become a key breakthrough to promoting digital inclusion. The perceived usefulness and perceived ease of use factors in the technology acceptance model provide perspective on the causes of older adults' negative attitudes toward technology. Strategies to overcome such attitudes can be categorized based on micro and macro policy paths. The latter could include establishing a community for the development of the eldercare service industry and focusing on age-friendly designs to improve the ease of use of these products. Community-based digital training services could help eliminate technology fears and promote social connection. Subdividing the demand market of older adults could lead to enhancing users' perceptions of the usefulness of the internet while paying attention to the special needs of vulnerable populations. Lowering the threshold for the use of information technology products could create a safe and green internet environment. Micro paths could include social workers providing digital training through new media teaching. As the objects of digital inclusion, older adults must have the right attitude to overcome their social exclusion. Their children must take a positive view of their potential to accept new things and become a strong backup for their use of technology products.

From a theoretical perspective, by establishing a localized digital inclusion measurement system, attitudes toward technology can be considered an influencing factor. Concerning internal connections among the digital inclusion of older adults, social networks, support, capital, and other factors, and how they jointly affect older adults' quality of life, are important Research Topics for further study.

Digital inclusion is assumed to be a good thing. Older adults who choose to stay away from the internet and technology will not be easily integrated into our rapidly evolving society. They risk a declining quality of life as the times leave them behind. Currently, older adults who do not know how to use internet technology products and services may have higher life satisfaction. This is likely because we are still in the process of moving toward a world of digital inclusion. The negative effects of having no access of technology are not yet absolute. With the advance of digital inclusion in a highly developed information age, measures must be taken to facilitate the information acquisition and social participation of these digital refugees. If nothing is done, the digital divide will become larger, affecting even social equity and the stability of society. We must actively pay attention to this issue. China vigorously promotes digital inclusion through public policy. By discussing a specific population, we can see digital inclusion in more detail. It is possible to look forward to a future in which digital connectivity joins different populations, regions, and industries and the digital divide is eliminated as much as possible.

## Data Availability Statement

The raw data supporting the conclusions of this article will be made available by the authors, without undue reservation.

## Ethics Statement

The studies involving human participants were reviewed and approved by Ethical Committee of School of Social Development and Public Policy in Fudan University. The participants provided their written informed consent to participate in this survey.

## Author Contributions

HY, HongtC, TP, and YL wrote the first draft. HonglC and YZ conducted the data collection and data analysis. All authors made revisions to the final manuscript.

## Funding

The study was jointly supported by China Social Academy Research Fund (Grant No. 21BSH130) and the Non-profit Central Research Institute Fund of Chinese Academy of Medical Sciences (Grant No. 2021-JKCS-026).

## Conflict of Interest

The authors declare that the research was conducted in the absence of any commercial or financial relationships that could be construed as a potential conflict of interest.

## Publisher's Note

All claims expressed in this article are solely those of the authors and do not necessarily represent those of their affiliated organizations, or those of the publisher, the editors and the reviewers. Any product that may be evaluated in this article, or claim that may be made by its manufacturer, is not guaranteed or endorsed by the publisher.
